# Collagen/Gelatin Sponges (CGSs) Provide Both Protection and Release of bFGF: An* In Vitro* Study

**DOI:** 10.1155/2019/4016351

**Published:** 2019-02-19

**Authors:** Maria Chiara Munisso, Naoki Morimoto, Sharon Claudia Notodihardjo, Toshihito Mitsui, Natsuko Kakudo, Kenji Kusumoto

**Affiliations:** Department of Plastic and Reconstructive Surgery, Kansai Medical University, Osaka, Japan

## Abstract

It has been reported that collagen/gelatin sponges (CGSs) are able to sustain the release of basic fibroblast growth factor (bFGF) for approximately 10 days via the formation of ion complexes between bFGF and gelatin. CGSs impregnated with bFGF have been proven to promote dermis-like tissue formation in various* in vivo* studies and clinical trials. However, the bioactivities of bFGF released from CGSs have not been explored* in vitro*. In this study, we explored the ability of CGS impregnated with bFGF, stored at 37°C for up to 14 days, to promote fibroblast proliferation and the sustained release of bFGF. We analyzed the cellular viability and proliferation in 2D and in 3D cell cultures, by a CCK-8 assay. Furthermore, in order to characterize the morphological alteration of fibroblasts, we studied 3D cultures by microscopy with a scanning electron microscope (SEM) and a confocal microscope. Our analyses revealed that the fibroblasts were elongated and flanked each other. They infiltrated and migrated inside the CGSs and were oriented along the CGS structure. Thus, these data prove that CGSs protect and sustain the efficient release of growth factor for more than 7 days.

## 1. Introduction

Wound healing is a complex, multicellular process aimed at epithelium restoration after injury. It relies on a multitude of growth factors and cytokines that tightly regulate a complex signaling network [1]. These biologically active molecules are present in the wound bed and play a role in all stages of wound healing: hemostasis, inflammation, proliferation and tissue remodeling [1, 2]. In the first stage, platelets play important roles, regulating primary hemostasis, and produce vasoactive mediators and chemotactic factors, such as proteases, cytokines, and growth factors [3]. Cytokines send out signals to inflammatory cells and local cell populations, and the fibrin–fibronectin clot serves as a temporary matrix to allow epithelial cells and fibroblasts to migrate into the wound [3]. Then, in the inflammatory phase, damaged and dead cells are cleared out and various growth factors and cytokines are released into the wound [4]. Several studies have demonstrated that this complex process is executed and regulated by a complex signaling network involving numerous growth factors, cytokines and chemokines. Of particular importance is the epidermal growth factor (EGF) family, transforming growth factor beta (TGF‐*β*) family, fibroblast growth factor (FGF) family, vascular endothelial growth factor (VEGF), granulocyte macrophage colony stimulating factor (GM-CSF), platelet‐derived growth factor (PDGF), connective tissue growth factor (CTGF), interleukin (IL) family, and tumor nerosis factor-*α* family [5]. Among all of the growth factors, basic fibroblast growth factor (bFGF) plays a crucial role in the wound healing process through the promotion of fibroblast proliferation, the induction of neovascularization, and the increased synthesis of collagenase [4, 6–8]. In the healthy healing wounds, fibroblasts are present from the late inflammatory phase until full epithelialization occurs. They are summoned to migrate to the wound area, proliferate, and carry out a number of key activities under the tight regulation of injury-mediated factors [9]. However, diabetes has an adverse effect on the proliferation of fibroblasts [10–12]. In fact, the most frequent causes of hospitalization in patients with diabetes are impaired tissue repair and foot ulceration [13].

In 2001, a wound healing product called Fiblast® Spray (Kaken Pharmaceutical Co., Ltd., Kyoto, Japan) was launched in Japan. This product contains recombinant human bFGF, which stimulates the proliferation of fibroblasts and promotes the formation of granulation tissue [14]. The topical administration of Fiblast Spray has been shown to be effective for the treatment of chronic skin ulcers [15–19] and deep wounds with exposed bone [20]. However, since the* in vivo* half-life of bFGF is very short, the daily administration of bFGF is required [19, 21]. Thus, in order to enhance the* in vivo* efficacy, bFGF has been incorporated in biodegradable sponges.

In this work, we have used collagen/gelatin sponges (CGSs). CGSs are composed of an outer silicone membrane and an inner collagen-gelatin sponge. The CGSs contain a 10wt% concentration of acidic gelatin that is capable of supporting the sustained release of positively charged growth factors such as bFGF [22]. The progressive infiltration of host cells, especially fibroblasts, stimulates the synthesis of new collagen fibers and the absorption of the CGSs. The CGS functions as a scaffold and spontaneously turns into autologous regenerated connective tissue, so-called dermis-like tissue, within 2 or 3 weeks. The silicone membrane is then peeled off and a secondary split-thickness graft is grafted onto the dermis-like tissue [22]. Previous studies have demonstrated that bFGF is released* in vivo* through the biodegradation of CGSs, and CGSs are impregnated with bFGF accelerate dermal tissue formation [6, 20, 22–26]. However, the effectiveness of CGS impregnated with bFGF* in vitro* has not been fully elucidated. Thus, the objective of this study was to evaluate the efficacy of CGSs impregnated with bFGF* in vitro*, in order to describe the basic processes involved in the associated wound healing.

## 2. Materials and Methods

### 2.1. Materials

The human recombinant bFGF (Fiblast® Spray 500) was purchased from Kaken Pharmaceutical, Tokyo, Japan. Two types of collagen/gelatin sponges (CGS, PELNAC Gplus®, Gunze Co., Ltd., Ayabe, Japan) were kindly supplied by Gunze Co., Ltd. All of the CGSs that were used were bilayered artificial dermis and consisted of an upper silicone sheet (thickness: 0.1 mm) and a lower sponge (thickness: 3 mm). In this study, CGSs with fenestrated type of PELNAC Gplus, rectangular shape: 15 mm × 20 mm and without fortified type of Pelnac Gplus, round shape: diameter 15.6 mm slits were used. The fenestrated type was used for the ELISA and the evaluation of* in vitro* cell proliferation (2D model). The fortified type was used for the evaluation of* in vitro* cell proliferation with CGS (3D model).

Trizma® base (#T1503), collagenase from* Clostridium histolyticum* Type IA (0.5-5.0 FALGPA units/mg solid, ≥125 CDU/mg solid, #C9891), was purchased from Sigma-Aldrich (St. Louis, Missouri, United States). Calcium chloride (#039-00475) and hydrochloric acid (0.1N Standardized Solution, #035644) were purchased from Fujifilm Wako Pure Chemical Corporation (Japan). Human dermal fibroblasts, adult (HDFa, #10407693), were purchased from Gibco (Fisher Scientific, Germany). Fibroblasts were cultured in DMEM (Dulbecco's modified medium Eagles medium, Nissui Pharmaceutical, # 05915) supplemented with 3.5 g D(+)glucose, 1.8 mg sodium bicarbonate (Wako Pure Chemical Industries Ltd., Osaka, Japan), 10,000 IU penicillin, and 10 mg streptomycin (penicillin-streptomycin; MP Biomedicals, LLC, Solon, OH, USA) and 10% FBS (HyClone™ Fetal Bovine Serum, #SH30910, Thermo Fisher Scientific Inc., United States).

### 2.2. Methods

#### 2.2.1. In Vitro Cell Proliferation (2D Model)

**Figure 1:**
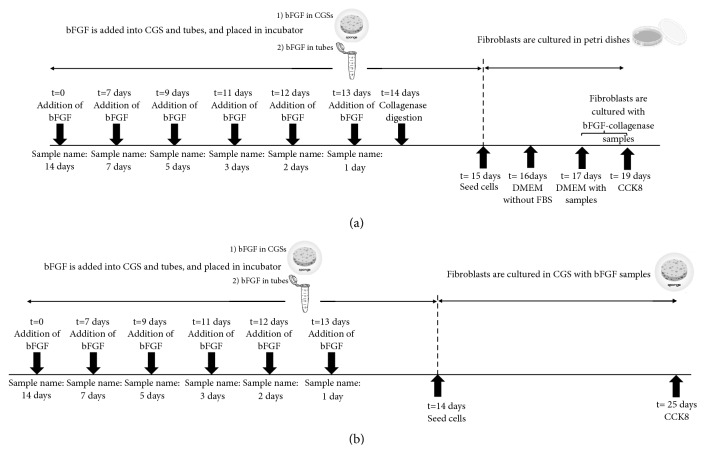
*A schematic illustration of the experimental procedure*. (a)* In vitro* cell proliferation (2D model) and (b)* In vitro* cell proliferation with CGS (3D model).

Human recombinant bFGF (500 *μ*g), in the form of a dry powder, was dissolved in 7.14 mL of normal saline solution (NSS, Otsuka Pharmaceutical Co., Ltd., Tokyo, Japan), and 70 *μ*g/mL of bFGF solution was prepared. Each CGS was placed in a 3.5 cm glass culture dish. Three hundred microliters of bFGF (7 *μ*g/cm^2^) was applied to each CGS and kept for 1, 2, 3, 5, 7, or 14 days at 37°C. The same volume of bFGF was added to small tubes (Eppendorf Protein LoBind Tubes) and placed inside the same incubator for the same amount of time. After incubation, each sample was suspended in a reaction mixture containing 10 U (3.8 mg) of collagenase in 4 mL of 50mM Tris/HCl containing 10 mM CaCl2 (pH 7.4, collagenase digestion buffer). Incubation was performed for 6 hours at 37°C in a cell incubator on a shaker (Iwaki Shaker mix SHM-100); then the liquid collected was analyzed by an ELISA (Human FGF basic Quantikine ELISA Kit (R&D Systems, Inc., Minnesota, United States) (Figure 1(a)).

Subsequently, in order to test the quality of the bFGF obtained, a small amount of the digested mixture was added to human fibroblast culture. A total of 3 x 10 ^3^ cells/well were seeded on a 96-well plate. After 24 h the medium was changed to DMEM without FBS. On the following day, different dilutions of samples obtained after collagenase digestion were added to the cultured fibroblasts. After 48 h, a cell proliferation assay was performed using a Cell Counting Kit-8 (CCK-8 kit, Dōjindo Laboratories, Kumamoto, Japan) (Figure 1(a)). Different concentrations of samples were studied (140, 35, and 8.75 ng/mL) both in the presence and in the absence of 2% FBS. The indicated concentrations (140, 35, and 8,75 ng/mL) are the theoretical value of bFGF, which was calculated as follows: 21 *μ*g bFGF in 300 *μ*L was added to each CGS; then additional 4mL of collagenase solution was added, in order to digest the CGS; thus, the final theoretical concentration of bFGF in the bFGF-collagenase solution was 0.0048*μ*g/*μ*L. Subsequently, before adding the samples to the fibroblast culture, we performed an extra dilution in DMEM (or DMEM + 2% FBS) to obtain the following bFGF theoretical concentrations: 140, 35, and 8.75 ng/mL.

The control samples used in these experiments were P3 (DMEM with 2% FBS), P2 (DMEM with 10% FBS), P1 (DMEM [or DMEM and 2% FBS] with fresh bFGF [the concentration was the same as the theoretical value in the bFGF-collagenase solution]), and Neg (DMEM without FBS).

#### 2.2.2. In Vitro Cell Proliferation with CGS (3D Model)

Twenty-two microliters of bFGF (0.4 *μ*g/cm ^2^) was added to each CGS in a 24-well plate, which was incubated for 1, 2, 3, 5, 7, or 14 days (Figure 1(b)). The same volume of bFGF was added to small tubes (Eppendorf Protein LoBind Tubes), placed inside the same incubator for the same amount of time, and then added on new CGSs 1 hour before fibroblasts seeding. A total of 4 x 10 ^4^ cells/well, in 100 *μ*L of 0.1% FBS - DMEM, were added to each CGS, and then incubated at 37°C. After 1.5 h DMEM with 0.1% FBS was added until the volume reached 500 *μ*L (P1, P2, P3, P4, and Neg control samples are described below). The medium was changed every 2 days, until 11 days of culture; then a cell proliferation assay was performed using CCK-8 (Figure 1(b)). Subsequently, 4% paraformaldehyde was added for 4 h at room temperature. The samples for SEM imaging were washed with saline 3 times and freeze-dried. The samples for confocal microscope imaging were treated, after permeabilization with 400 *μ*L of 0.1% triton for 5 min, with 400 *μ*L of Rodamine X-conjugated phalloidin (# 165-21641 Fujifilm Wako Pure Chemical Corporation, Japan) for 30 min, and then with 400 *μ*L of Hoechst solution (bisBenzimide H 33342 trihydrochloride, #B2261, Sigma- Aldrich, St. Louis, Missouri, United States) for 15 min. After every step, the samples were washed 3 times with saline.

The control samples used in these experiments were as follows: P4 (0.1% FBS-DMEM); P3 (2% FBS-DMEM); P2 (10% FBS- DMEM); P1 (DMEM [or DMEM and 2% FBS] with fresh bFGF [the concentration of bFGF was the same as the theoretical value in the bFGF-collagenase solution]) and Neg (DMEM without FBS).

#### 2.2.3. Statistics and Data Analysis

Analyses between groups were made using a 1-way ANOVA followed by a Turkey for multiple comparisons (GraphPad Prism 7; La Jolla, CA). All data are expressed as the mean ± standard deviation. A P value of <0.05 was accepted as being statistically significant.

## 3. Results 

**Figure 2:**
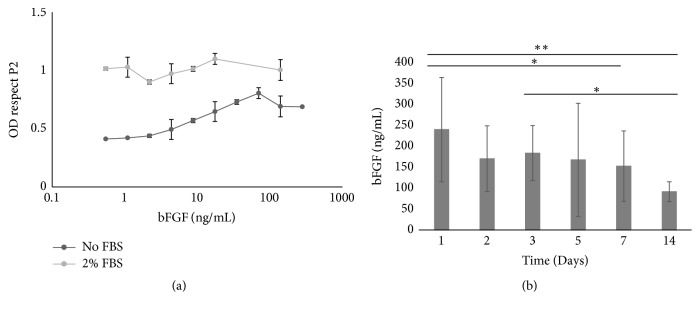
*The effect of the bFGF concentration*. (a) The effect of the bFGF concentration on the fibroblast proliferation in DMEM or DMEM supplemented with 2% FBS is reported. The results are expressed as a ratio of the optical density (OD) values of each samples to the OD results of control sample P2 (samples treated with DMEM with 10%FBS). (b) The bFGF content determined by an ELISA assay (N=5) in samples kept at 37 degrees for 1, 2, 3, 5, 7, and 14 days. 1-way ANOVA, Tukey's multiple comparisons test. *∗∗* p<0.001 and *∗* p<0.01.

Fibroblasts were cultured with different concentrations of bFGF in presence and absence of 2% FBS. Our results showed that the presence of 2% FBS may mask the effect of small quantities of bFGF added to cell medium. Instead, in the absence of FBS, the number of cells increased with increasing amounts of bFGF until reaching a plateau (Figure 2(a)).

The same amount of bFGF was added to CGSs, maintained at 37°C for several days, and then digested with collagenase solution. The quantitative measurement of bFGF contained in the digested solutions was performed using a human FGF basic ELISA kit, according to the manufacturer's instructions, in order to evaluate the retention of bFGF. The quantity of binding bFGF on CGSs was calculated, and no significant differences in bFGF content were found in the first 5 days (Figure 2(b)). A clear and significant difference was observed between day 1 and days 7 and 14.

### 3.1. In Vitro Cell Proliferation (2D Model)

**Figure 3:**
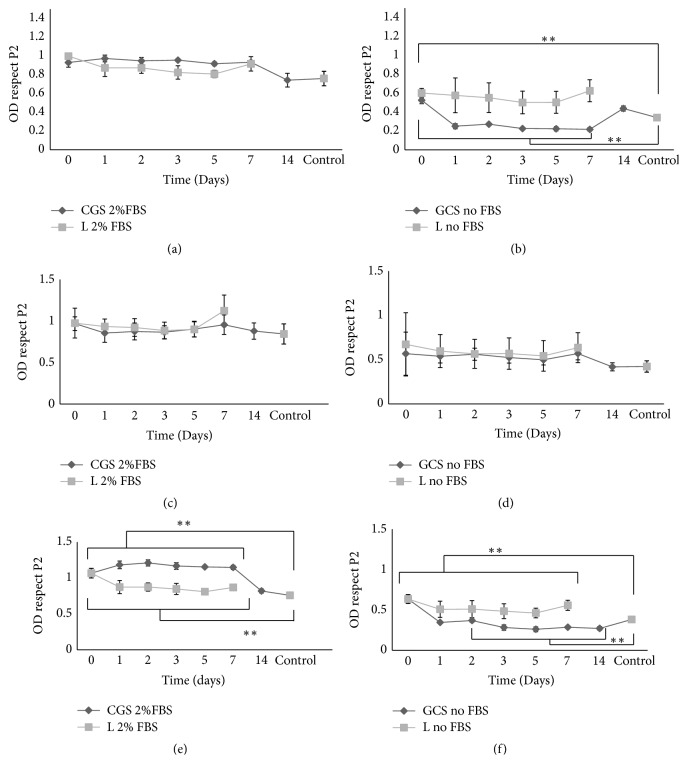
*The effect of the bFGF concentration on fibroblast proliferation*. Proliferation of fibroblasts at (a)-(b) 140ng/mL, (c)-(d) 8.75 ng/mL, and (e)-(f) 35 ng/mL, in the absence of 2% FBS and (d) in the presence of FBS, respectively. CGS indicates that bFGF was added to CGS; L indicates that bFGF was added to small tubes and kept under the same conditions for the same amount of time. 2% FBS indicates that 2% FBS was added to the culture media; no FBS indicates that FBS was not added to the culture media. For samples in presence of FBS, control sample is P3, DMEM + 2% FBS. For samples in absence of FBS, control sample is Neg, DMEM. (N=6). The results are expressed as a ratio of the optical density (OD) values to the OD results of control samples P2. 1-way ANOVA, Tukey's multiple comparisons test. *∗∗* p<0.001 and *∗* p<0.01.

Different concentrations of digested samples, both in the presence and absence of 2% FBS, were studied: 140 (Figures 3(a)-3(b)), 35 (Figures 3(e) and 3(f)) and 8.75ng/mL (Figures 3(c) and 3(d)). At 140 ng/mL, in the absence of FBS, the fibroblast proliferation was significantly lower than the proliferation of the control (Neg, DMEM no FBS) (Figure 3(b)-CGS no FBS). This indicated that the collagenase present in the solution damaged the fibroblasts. After the addition of 2% FBS (Figure 3(a)-CGS 2% FBS), the proliferation was higher than control (P3, DMEM with 2% FBS). However, the proliferation was not significantly different from liquid bFGF kept for the same amount of time at 37°C in small tubes (Figures 3(a) and 3(b)-L 2% FBS and L no FBS). Figures 3(c) and 3(d) show the results for 8.75ng/mL. The quantity of bFGF added to the fibroblasts was too low to observe a clear trend; however, it was evident that 8.75ng/mL contained less collagenase and had a very small effect on cell damage (Figure 3(d)-CGS noFBS). Figures 3(e) and 3(f) show the results for 35ng/mL. The results show that, in the absence of FBS, collagenase damaged the fibroblasts; in fact the OD values of samples kept for 3, 5, 7, or 14 days were lower than control (Figure 3(f)-CGS no FBS), while samples kept for 1 or 2 days were similar. This seems to indicate that the presence of bFGF in these two samples mitigated the negative effects of collagenase but was not enough to promote proliferation. Moreover, when liquid bFGF was kept at 37°C for 1 or 2 days, the activity of bFGF was not completely destroyed (Figure 3(f)- L no FBS). In fact, the activity of the samples (L no FBS) on days 1 and 2 was higher than control. In the presence of 2% FBS, it is clear that GSCs are able to protect the bFGF from the environment (Figure 3(e)-CGS 2% FBS) until day 7.

### 3.2. In Vitro Cell Proliferation with CGS (3D Model)

**Figure 4:**
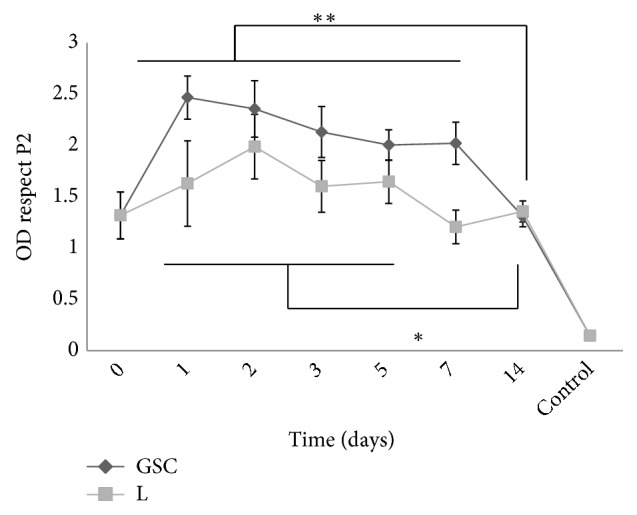
*The effects of the bFGF concentration on fibroblast proliferation in CGS*. CGS indicates that bFGF was added to CGS. Liquid indicates that bFGF was added to small tubes and kept under the same conditions. x indicates the number of days. Control samples P 4 indicated samples treated with DMEM + 0.1% FBS (N=6). The results are expressed as a ratio of the optical density (OD) values to the OD results of control samples P2 (samples treated with DMEM with 10%FBS). 1-way ANOVA, Tukey's multiple comparisons test. *∗∗* p<0.001 and *∗* p<0.01.

**Figure 5:**
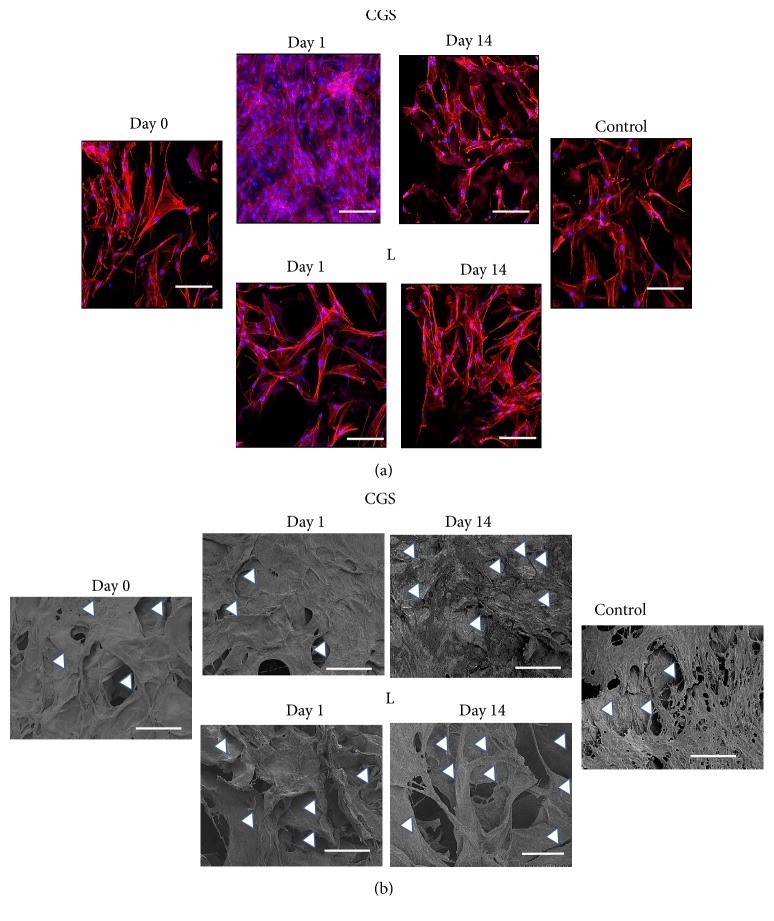
*Confocal fluorescence microphotographs and SEM images*. (a) Confocal fluorescence microphotographs of 6 representative samples. Phalloidin (red)/Hoechst (blue). (b) SEM images. CGS indicates that bFGF was added to CGS. L indicates that bFGF was added to small tubes and kept under the same conditions. Control samples: P4, DMEM + 0.1% FBS. In Supplementary Figure 2 the images for all the samples are reported. Scale bar is 50 *μ*m. White arrowheads indicate the CGS.

CGSs impregnated with bFGF were kept at 37°C for 1, 2, 3, 5, 7, or 14 days. Then fibroblasts were seeded and allowed to grow for 11 days (Figure 1(b)). In the samples kept at 37°C for 7 days, the number of cells cultured in CGSs impregnated with bFGF (Figure 4-CGS) was significantly higher than the number of cells cultured in CGSs impregnated with liquid bFGF kept at 37°C (Figure 4-L).  Figure 5 shows representative fluorescence and SEM images of adherent cells cultured on CGS. In the fluorescence images, actin cytoskeletons and nuclei are shown in red and blue, respectively (Figure 5). Images collected from all the samples are in Supplementary Figure 2.

## 4. Discussion

Since bFGF has a short half-life, to improve its administration to patients without daily treatement, it was incorporated in CGSs. Tabata et al. [27] studied the sorption of bFGF into gelatin hydrogel in two different liquid: phosphate-buffered saline (PBS) and 1.5N NaCl. In addition, they analyzed the time profile of bFGF sorption to the acidic gelatin hydrogel and showed that it depended on the solution temperature and approximately 10% of bFGF sorbed was desorbed in PBS within the initial 1 h [27]. bFGF has a theoretical isoelectric point of 9.58 and thus has a positive charge around physiological pH, so it can be incorporated into scaffolds by noncovalent interaction. This allows a sustained release of bFGF [6, 20, 22–26]. In our experiments, bFGF was added to CGS and kept at 37°C for up to 14 days. The CGS was then destroyed by collagenase and the liquid collected was analyzed by an ELISA to determine the bFGF content. Subsequently, the liquid collected from each sample was added in small quantities to fibroblast cultures in order to determine the bFGF activity (Figure 1(a)). In fact, active bFGF will also promote fibroblast growth in the absence of heat-inactivated fetal bovine serum (FBS). The main problem with this protocol is that the solution contains active collagenase. Collagenase is a common enzymatic agent use to passage or harvest cells. Although collagenase treatment has been reported to be the least traumatic harvesting method [28], the addition of a small amount of active collagenase to fibroblast cultures affected cell growth. In Supplementary Figure 1, the cell growth with the increasing quantities of collagenase and bFGF is reported. In absence of FBS the cell growth decreases almost linearly with the increasing of the collagenase contain. Instead when 2%FBS is added to the solution, a similar trend to the one shown in the experimental data in Figure 2(a) was observed. There is an increase of cell growth and then at higher concentration of bFGF a decrease. This clearly confirms the detrimental effect of the collagenase in absence of FBS in the cell media in the 2D experiments design.

However, it is well known that collagenase activity can be inactivated with FBS; thus, we investigated whether the addition of 2% FBS had a concentration-dependent effect on fibroblast growth in the presence of bFGF. In fact, 2% FBS was sufficient to inactivate the collagenase; however, it might influence the growth of cells and hide the effects of bFGF. Our results (Figure 2(a)) showed that the presence of 2% FBS masked the effects of small quantities of bFGF added to cell medium. Moreover, the addition of 2% FBS had a much greater effect on cultures with very low bFGF content. Nevertheless, it had a small effect on cultures with intermediate bFGF concentrations. Based on this particular behavior, we decided to perform all the experiments in the presence and absence of 2% FBS, in order to separate the effects of FBS from the effects of bFGF.

We previously confirmed that CGS could sustain bFGF and release it according to its biodegradation in a sustained manner* in vivo* and* in vitro *[22, 23, 25, 29]. In particular, previous release profile studies in PBS suggest that the release of bFGF from CGS involves two different processes. At first, in PBS solution, bFGF is released mainly due to the effects of simple diffusion at first. Then, bFGF ionically adsorbed to gelatin molecules is released via the enzymatic degradation of the gelatin operated by the collagenase [23]. The quantitative measurement of bFGF by an ELISA did not detect a significant difference in the bFGF content over a 7-day period, while a clear difference was observed after 7 days. However, from the experimental results, it seems that the concentration of bFGF is lower than the expected values. Several causes can determine these results. First of all, the ELISA method involves comparison of test samples to a standard curve prepared using known concentrations of the analyte (e.g., purified recombinant protein). The goal in assay is to maximize signal-to-noise ratio while achieving identical responses for a given amount of analyte in the standard diluent (the standard curve) and sample. However, samples may contain components that affect assay response. So, before start the experiments it is necessary to control the method specificity, linearity, accuracy, precision, range, limit of detection, and limit of quantitation. Therefore, the first step in our experiments was to verify these parameters. We found that the sensitivity and detection limit change with the diluents used. Although they are lower when a solution with collagenase is used, accuracy, precision, and linearity were verified also using a solution with collagenase. In addition, the lower value of bFGF detected by ELISA can be caused by other important factors, such as the short half-life of bFGF in the environment. Moreover, part of the bFGF might bind to tips and other material used in the experiment. Though, all these factors are affecting our results decreasing the detected bFGF quantity; the final results have high reproducibility and accuracy.

### 4.1. In Vitro Cell Proliferation (2D Model)

The ELISA results (Figure 2(b)) were compared with the fibroblast proliferation after the addition of small amount of digested solutions (Figure 3). In our experiments the concentration of bFGF was decided according the results of the experiments reported in Figure 2(a) and in Supplementary Figure 1. In Figure 2(a), it is possible to see that the increasing of bFGF concentration can improve the proliferation of fibroblast till a maximum value and then starts to decrease. In the experiments the mix of digested sponges was added on the cell cultures. Thus, a mix of bFGF and collagenase, which will affect the proliferation of cells, was added. In Supplementary Figure 1, it is possible to see that 35 ng/mL is one of the 3 concentrations of bFGF with higher OD (so higher cell proliferation), but it is the only concentration with low collagenase solution. So, this concentration was selected to be the most representative condition for our experimental set up. In fact, the scattered fibroblast proliferation after the addition of 8.75ng/mL (Figures 3(c) and 3(d)) of bFGF showed that the bFGF value was too low. In contrast, when 140 ng/mL (Figures 3(a) and 3(b)) was added in the presence of 2% FBS, a difference was observed between the CGS and the liquid samples. However, the quantity of collagenase in the solution without FBS was too high; thus, we could not confirm the trend. Figures 3(e) and 3(f) show the results for 35ng/mL. In these latest figures (Figures 3(e) and 3(f)) a clear difference between the CGS and the liquid samples was observed in both the presence and the absence of FBS. Moreover, it is important to notice that the CGSs are able to preserve the activity of bFGF for 7 days.

### 4.2. In Vitro Cell Proliferation with CGS (3D Model)

With regard to the types of CGSs, the fenestrated type is most commonly used in clinical practice because it has many slits to allow for the drainage of exudate or blood from the wound. Thus, we used the fenestrated type for the ELISAs and the evaluation of* in vitro* cell proliferation (2D model). However, in the 3D model, we used the fortified type without slits because the effect of the slits on fibroblast morphology and proliferation has not been demonstrated. We would like to clarify that the aim of this study is not to explain differences between fenestrated and fortified type CGSs. This type of study required additional experiments and it will be done in future. When fibroblasts were seeded on CGSs (Figure 4) the cell growth was significantly higher in CGSs impregnated with bFGF and stored until day 7 day at 37°C. In the fluorescence images (Figure 5(a)), the formation and growth of actin stress fibers showed that the cells adhered to the CGSs. These results confirmed that fibroblasts could infiltrate and migrate inside the CGS from the surface (the top of the CGS, where fibroblasts were seeded) until the silicon layer (the bottom of the CGS). In the bFGF-impregnated CGSs the cell density was very high, and it was difficult to distinguish the cells from the CGS structure on SEM images, especially on day 1 (Figure 5(b)-CGS). Instead, in the CGSs impregnated with liquid that was kept at the same condition of the impregnated CGSs, it is possible to see the porous GCSs structure (Figure 5(b)-L).

The present study quantified the bFGF activity in 2D and 3D cultures after incubation for 1, 2, 3, 5, 7, or 14 days. The comparison of the data obtained from 2D and 3D cultures indicates that it is possible that (i) bFGF in CGS maintains its activity until day 7, (ii) there is little change in the bFGF activity in 2D cultures in the first 7 days of incubation, and (iii) the bFGF activity in 3D cultures slowly decreases from day 1 to day 14. The difference between 2D and 3D cultures is due to two fundamental events: protection and controlled release. In 2D cultures, fibroblasts were cultured for 2 days in medium supplemented with a solution of bFGF (Figure 2(b)), and we observed that bFGF (in impregnated CGS) maintained its activity after incubation at 37°C for 7 days, after which its activity drastically decreased (Figure 4). This demonstrated that CGS is able to protect the bFGF from the environment. In 3D culture, it was possible to observe a combination of protection (CGS protects the bFGF from the environment for 7 days) and controlled release over the additional 11 days of cell culture. Thus, the 3D culture experiment confirmed that CGSs can protect bFGF for 7 days and and sustain the efficient release of growth factor over a period of at least 11 days.

## 5. Conclusions

The present study showed the effectiveness of bFGF-impregnated CGSs* in vitro*. CGSs were able to protect the bFGF until day 7 at 37°C. Moreover, the 3D cultures of fibroblasts on bFGF-impregnated CGSs proved the protective effect of the CGS and its ability to improve the controlled release over a period of 11 days.

## Data Availability

The data used to support the findings of this study are included within the article and the supplementary information file(s).

## Supplementary Materials

Supplementary Materials Supplementary Figure 1: The effect of the bFGF concentration on fibroblast proliferation. Proliferation of fibroblasts at different concentration of bFGF in the absence and in the presence of FBS. CGS indicates that bFGF was added to CGS (N=6). Supplementary Figure 2. Confocal fluorescence microphotographs and SEM images. Phalloidin (red)/ Hoechst (blue). CGS-x indicates that bFGF was added to CGS and x indicated the number of days. L-x indicates that bFGF was added to small tubes and kept under the same conditions. x indicates the number of days. Control samples: P2, DMEM + 10% FBS; P3, DMEM + 2% FBS; P4, DMEM + 0.1% FBS; Neg, DMEM. Samples: DMEM + 0.1% FBS.
