# Supplementary material for: Collagen/Gelatin Sponges (CGSs) Provide Both Protection and Release of bFGF: An In Vitro Study
Source: Biomed Res Int. 2019 Feb 19;2019:4016351. doi: 10.1155/2019/4016351 (PMC6399556; doi:10.1155/2019/4016351)
Supplement: Supplementary Materials — Supplementary Figure 1: The effect of the bFGF concentration on fibroblast proliferation. Proliferation of fibroblasts at different concentration of bFGF in the absence and in the presence of FBS. CGS indicates that bFGF was added to CGS (N=6). Supplementary Figure 2. Confocal fluorescence microphotographs and SEM images. Phalloidin (red)/ Hoechst (blue). CGS-x indicates that bFGF was added to CGS and x indicated the number of days. L-x indicates that bFGF was added to small tubes and kept under the same conditions. x indicates the number of days. Control samples: P2, DMEM + 10% FBS; P3, DMEM + 2% FBS; P4, DMEM + 0.1% FBS; Neg, DMEM. Samples: DMEM + 0.1% FBS. [file 4016351.f1.pdf]

Supplementary materials

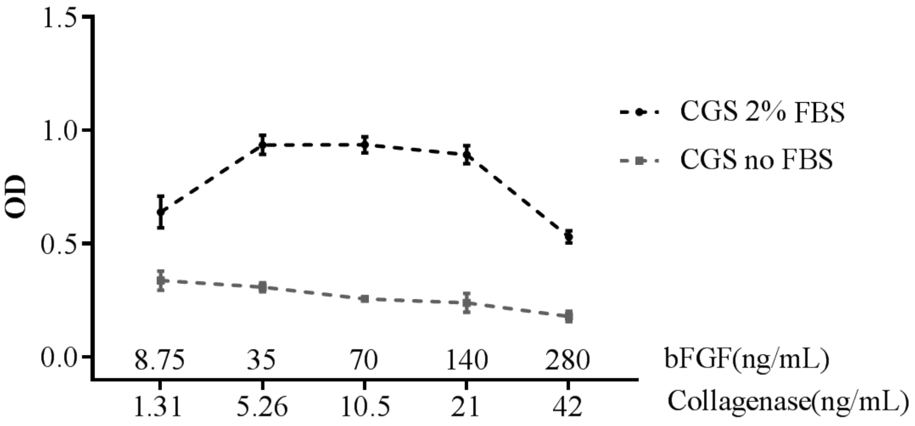

Supplementary Figure 1: The effect of the bFGF concentration on fibroblast proliferation. Proliferation of fibroblasts at different concentration of bFGF in the absence and in the presence of FBS. CGS indicates that bFGF was added to CGS (N=6).

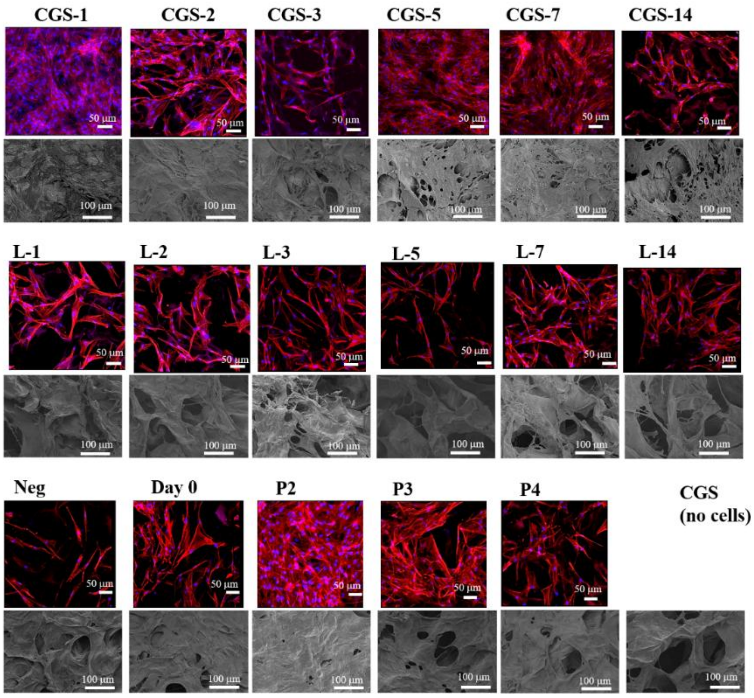

Supplementary Figure 2. Confocal fluorescence microphotographs and SEM images. Phalloidin (red)/ Hoechst (blue). CGS-x indicates that bFGF was added to CGS and x indicateds the number of days. L-x indicates that bFGF was added to

small tubes and kept under the same conditions. x indicates the number of days. Control samples: P2, DMEM + 10% FBS; P3, DMEM + 2% FBS; P4, DMEM + 0.1% FBS; Neg, DMEM. Samples: DMEM + 0.1% FBS.
